# Favorable Outcome With Early Vitrectomy for Pseudomonas Endophthalmitis Following Elective Pars Plana Vitrectomy for Disabling Vitreous Opacities

**DOI:** 10.1155/crop/4008092

**Published:** 2026-10-07

**Authors:** Roselind L. Ni, Evelyn G. Miller, Jonathan Perez, Allen Chiang, Michael A. Klufas

**Affiliations:** ^1^ Retina Service of Wills Eye Hospital Mid Atlantic Retina Thomas Jefferson University Philadelphia Pennsylvania USA jefferson.edu; ^2^ Sidney Kimmel Medical College Thomas Jefferson University Philadelphia Pennsylvania USA jefferson.edu; ^3^ Sargent College of Health & Rehabilitation Sciences Boston University Boston Massachusetts USA bu.edu

**Keywords:** endophthalmitis, pars plana vitrectomy, postoperative endophthalmitis, *Pseudomonas aeruginosa*, vitrectomy for vitreous floaters

## Abstract

Postvitrectomy endophthalmitis is a rare but vision‐threatening complication, with reported incidence rates below 0.15%, and treatment guidelines remain limited. Evidence guiding the timing of repeat vitrectomy in this setting is sparse, and the approach to management is derived largely from postcataract endophthalmitis. *Pseudomonas aeruginosa* endophthalmitis is associated with characteristically poor visual outcomes, often including enucleation. A 58‐year‐old man with a history of insulin‐dependent diabetes and proliferative diabetic retinopathy presented with conjunctival injection, corneal edema, hypopyon, and vitritis 2 days after elective 25‐gauge pars plana vitrectomy for vitreous opacities. His visual acuity had dropped from a preoperative baseline of 20/30 to hand motion. The patient was initially treated with vitreous tap and intravitreal injection of vancomycin and amikacin at initial presentation of endophthalmitis and admitted for hourly topical moxifloxacin and prednisolone acetate 1%. Re‐evaluation after 12 h showed persistently decreased vision and inflammation, with B‐scan ultrasonography showing active vitritis without retinal detachment. He was taken for repeat vitrectomy with intravitreal antibiotic injection within 24 h of presentation. Vitreous culture was positive for *Pseudomonas aeruginosa*, which was susceptible to the empiric aminoglycoside. The patient′s vision improved to 20/200 at 2 weeks, then to his baseline of 20/30 by 1 year postoperatively, remaining stable through a 3‐year follow‐up period. Early repeat vitrectomy within 24 h of endophthalmitis presentation and prompt intravitreal antibiotic therapy for patients with postvitrectomy gram‐negative endophthalmitis can be associated with favorable visual outcomes. Early patient presentation, concordant antibiotic treatment, and absence of severe complications, such as retinal detachment or occlusive vasculitis, may have contributed to excellent recovery to baseline vision.

## 1. Introduction

Endophthalmitis, characterized by severe inflammation of ocular tissues, is an ocular emergency due to its rapid and destructive nature, with patients typically presenting with pain, hypopyon, and dense vitritis [1]. Acute‐onset postoperative infectious endophthalmitis is the most common cause [1]. Postvitrectomy endophthalmitis is a rare but serious and vision‐threatening complication of pars plana vitrectomy (PPV). The incidence of post‐PPV endophthalmitis is low, ranging from 0.03% to 0.14% for 20G PPV [1]. Cases of endophthalmitis following cataract surgery and intravitreal injection are much more commonly reported, with incidence rates varying from 0.07% to 0.4% and 0.038% to 0.065%, respectively [1].

Historically, visual outcomes after post‐PPV endophthalmitis are poor with little posttreatment visual gain, despite early intervention [1]. Outcomes are typically worse than those after cataract surgery, which may be due to underlying vitreoretinal diseases for which vitrectomy was indicated [1]. Postoperative endophthalmitis is usually caused by gram‐positive bacteria, with coagulase‐negative staphylococci reported as the most common causal organism [1]. Gram‐negative infections are relatively rare but can cause fulminant destruction because of their high virulence and often occlusive vasculitis [2]. The outcomes of endophthalmitis caused by *Pseudomonas aeruginosa* are extremely poor [3].

Limited studies and guidelines exist for early repeat vitrectomy in acute post‐PPV endophthalmitis, particularly after elective vitrectomy. We present a case of acute infectious endophthalmitis secondary to *Pseudomonas aeruginosa* following elective PPV for vitreous opacities. Despite a severe gram‐negative infection, an initial vitreous tap and injection of antimicrobial agents followed by an early repeat vitrectomy may have contributed to an excellent anatomic and final visual acuity (VA) outcome.

## 2. Case Presentation

A 58‐year‐old attorney and former smoker with insulin‐dependent diabetes, ocular hypertension, early glaucoma, and bilateral proliferative diabetic retinopathy (PDR) status post panretinal photocoagulation presented for an elective PPV to address disabling vitreous floaters in the left eye. The floaters were remnants of prior vitreous hemorrhage from PDR and impaired his ability to complete work‐related visual tasks.

Preoperatively, optical coherence tomography (OCT) demonstrated a preserved foveal contour OU, with foveal thicknesses of 266 *μ*m OD and 270 *μ*m OS (Figure 1A). Prior to surgery, the patient had an HbA1c of 7.3. Intraocular pressure (IOP) was 15 mmHg, and VA was 20/30. The patient underwent 25‐gauge PPV with air‐fluid exchange without complications.

**Figure 1:**
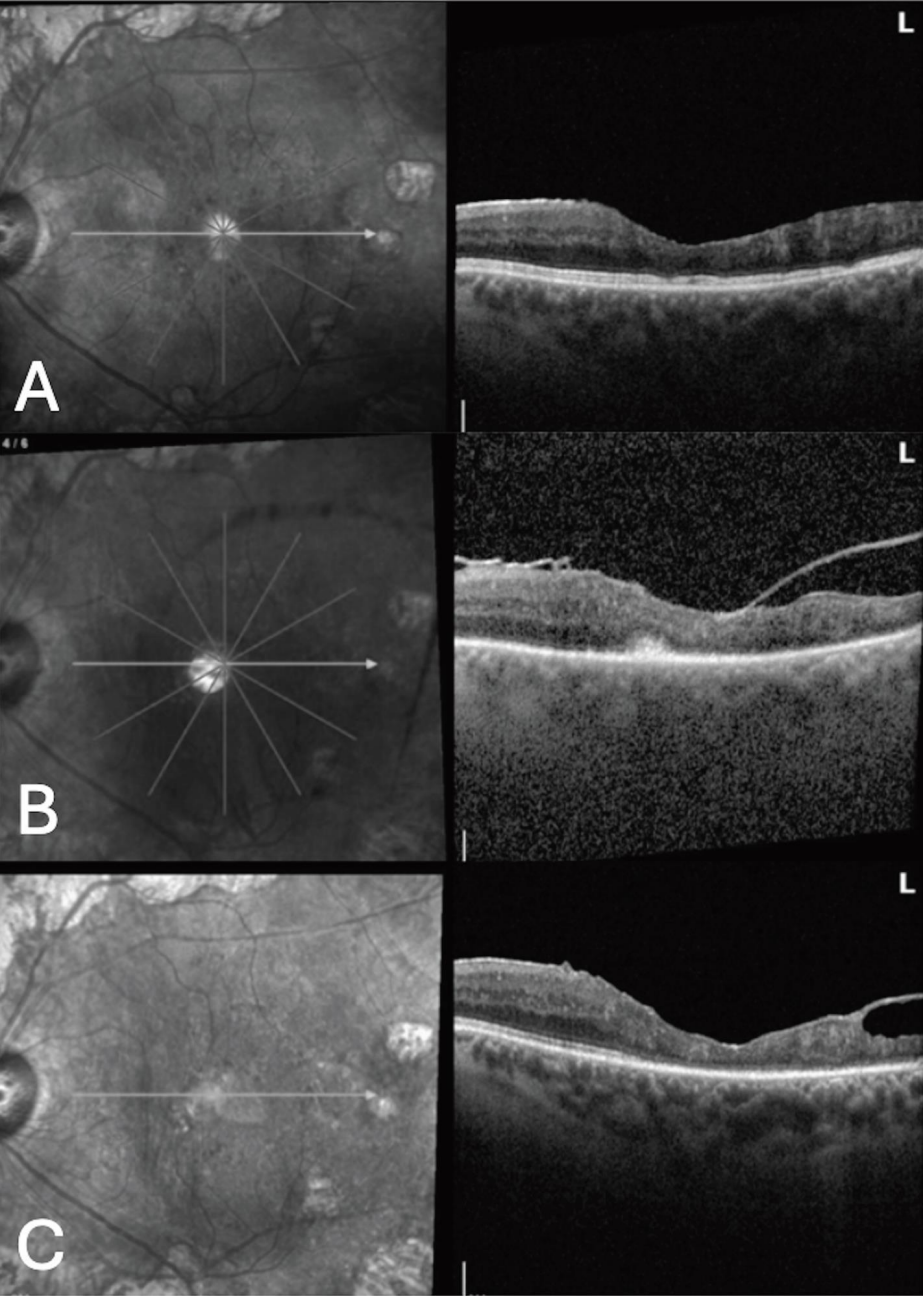
(A) Optical coherence tomography of the left eye before initial vitrectomy with normal foveal contour, (B) 2 weeks after repeat vitrectomy for endophthalmitis, with development of a preretinal ERM, and (C) 2.5 years postoperatively showing an attached retina with preserved foveal contour and mild stable ERM.

The patient had a history of sleep apnea and resumed continuous positive airway pressure (CPAP) the night after surgery. On postoperative Day 1, the patient reported no complaints, and exam showed no signs of inflammation. On the morning of postoperative Day 2, 48 h post‐PPV, he returned with severe pain and hypopyon, and his VA was hand motion. IOP was 20 mmHg. Exam showed conjunctival injection, corneal edema, > 1 mm hypopyon, and dense vitritis. At this visit, vitreous tap and intravitreal injection of vancomycin (1 mg/0.1 mL) and amikacin (0.4 mg/0.1 mL) were performed. The patient was then admitted for hourly topical antibiotic (moxifloxacin) and steroid (prednisolone acetate 1%) treatment and closely monitored.

Upon reassessment 12 h after the vitreous tap and injection, the patient reported improved pain but persistent decreased vision. B‐scan ultrasonography at this time showed the presence of vitreous debris consistent with active vitritis, without evidence of retinal detachment. Given persistent inflammation and good VA prior to infection, the decision was made to proceed with early repeat vitrectomy in the morning. Repeat 25‐gauge PPV with anterior chamber washout, partial fluid air exchange, and repeat injection of vancomycin (1 mg/0.1 mL) and amikacin (0.4 mg/0.1 mL) was performed 24 h after initial presentation of endophthalmitis. The retina was attached with good panretinal photocoagulation. Preretinal fibrinous material was noted, but there was no occlusive retinal vasculitis. Vitreous sample was culture positive for *Pseudomonas aeruginosa*, confirming gram‐negative infectious endophthalmitis. Antibiotic susceptibility testing showed that the causal pathogen was sensitive to quinolones and aminoglycosides. One week postvitrectomy, pain and inflammation had improved. At Week 2, VA improved to 20/200. OCT revealed the development of a preretinal ERM, followed with observation (Figure 1B).

The patient′s VA recovered to 20/30 by 1 year postoperatively, returning to his baseline, and remained stable at 20/30 throughout a 3‐year follow‐up period. At 2.5 years, OCT showed an attached retina with preserved foveal contour and mild residual ERM (Figure 1C), and IOP remained well‐controlled at 20 mmHg OS. The patient continued brimonidine tartrate 0.1% drops for pre‐existing glaucoma, and lifitegrast 5% drops for dry eye.

## 3. Discussion

In this report, we describe excellent visual recovery in a 58‐year‐old diabetic patient after he was treated with early repeat vitrectomy 24 h after initial endophthalmitis presentation for acute *Pseudomonas* endophthalmitis following an elective vitrectomy for vitreous opacities.

Risk factors for post‐PPV endophthalmitis include type of intraocular tamponade and contamination of the vitreous cavity [1]. Our patient underwent 25‐gauge PPV. Early reports raised the concern that sutureless small‐gauge sclerotomies may introduce bacterial entry and increase endophthalmitis rates. However, subsequent large studies found no significant difference in rates across gauge sizes [4–6]. Diabetes is a major risk factor for postoperative endophthalmitis, increasing infection risk because of relative immunocompromise [1]. Our patient had an elevated HbA1c of 7.3 prior to surgery, indicating suboptimal glycemic control. Our patient also resumed CPAP postoperatively; however, infection risk of CPAP use has been rarely studied and poorly understood [7]. *Pseudomonas aeruginosa* and other gram‐negative species have been isolated from CPAP machines, but there is no known association with increased infection risk [7].

*Pseudomonas aeruginosa* frequently leads to poor outcomes even with timely treatment. Outcomes of *Pseudomonas* endophthalmitis have been well reported, but there is a lack of data assessing its treatment with early vitrectomy. Chen et al. (2011) analyzed 72 eyes with *Pseudomonas* endophthalmitis, and although timing was undefined, 5 of 16 eyes (31%) treated with vitrectomy and intravitreal antibiotics achieved VA of 5/200 or better compared with only 1 of 45 eyes (2%) treated with tap‐and‐inject alone [8]. The remaining 11 eyes (15.3%) required evisceration [8]. Although few studies stratify outcomes based on treatment approach, data have shown consistently poor visual prognosis. Eifrig et al. (2003) analyzed 28 eyes with *Pseudomonas* endophthalmitis, all receiving intravitreal antibiotics with PPV performed in 12 eyes (43%) and found that 18 eyes (64%) underwent evisceration or enucleation, including seven (25%) as initial treatment [9]. Only two eyes (7%) achieved a final VA of 5/200 or better [9]. Subsequent studies have demonstrated poor final vision of 20/400 or worse in over 90% of patients and evisceration or enucleation in nearly half of eyes [3, 10].

Because of its rare incidence, management of post‐PPV endophthalmitis is not well studied. The landmark *Endophthalmitis Vitrectomy Study* (EVS) published in 1995 has strongly influenced protocols in treatment, concluding vitreous tap and biopsy to be equivalent to PPV in treating eyes with vision better than light perception [11]. However, several factors limit the applicability of the study to our case. The EVS included only postcataract endophthalmitis, excluding postvitrectomy cases [11]. Gram‐negative organisms were also significantly underrepresented, as only 6% of the 323 confirmed culture isolates were gram‐negative [11]. Only 4 out of the 420 patients included had *Pseudomonas* endophthalmitis [11]. Additionally, the EVS was conducted using 20‐gauge equipment. Modern small‐gauge vitrectomy produces less vitreoretinal traction and tissue trauma than the 20‐gauge vitrectomy, and advances in intraoperative visualization and cutting speeds likely allow for safer and more efficient surgery in the setting of infection [2].

Intravitreal antibiotic injection remains the mainstay of initial treatment. The timing of vitrectomy remains a subject of debate; however, its earlier use has increasingly been adopted. Welch et al. (2022) examined 290 patients with endogenous endophthalmitis or endophthalmitis following cataract surgery, intravitreal injection, vitrectomy, glaucoma surgery, corneal surgery, or nonsurgical trauma, and found that a planned early vitrectomy within 24 h of presentation resulted in better visual outcomes at 9 months compared with primary vitreous tap and injection [12]. Similarly, Blom et al. (2023) analyzed 41 cases of endophthalmitis following either injection, cataract surgery, vitrectomy, or blebi‐related surgery. Their study found that the majority of patients receiving immediate vitrectomy within 6 h or primary intravitreal antibiotics and early vitrectomy within a mean time of 23 h achieved clinically meaningful improvements in VA [13]. However, the results from these past studies should be interpreted with a few considerations in mind. They included endophthalmitis of heterogeneous etiology, nonrandomized treatment allocation, variable timing of surgery, and selection bias, as the choice of vitrectomy was likely influenced by presenting severity of disease.

Our report illustrates a favorable outcome following early intravitreal antibiotics at initial presentation and early repeat vitrectomy within 24 h of presentation in returning a diabetic patient′s vision back to baseline after infection by *Pseudomonas aeruginosa*, which typically causes poor outcomes including enucleation. Limitations of this case report include its retrospective nature, small sample size of a single case, and limited generalizability. The favorable outcome in our case must be interpreted in the context of potential confounding factors. Our patient presented within 24 h of symptom onset, which enabled prompt initiation of treatment before extensive damage. The causative organism was also found to be susceptible to aminoglycosides, and amikacin was included in his empiric antibiotic treatment. Thus, appropriate antibiotic coverage was achieved from the onset of endophthalmitis. *Pseudomonas aeruginosa* is known for its ability to develop multidrug resistance, and cases treated with antibiotics lacking concordant susceptibility likely suffer worse outcomes. Additionally, the absence of severe complications of endophthalmitis, such as retinal detachment or occlusive vasculitis, may have been associated with our patient′s visual recovery [14].

Our patient was followed for 3 years after initial elective PPV and maintained stable 20/30 vision. Although prevention remains paramount, this report shows that early PPV may be associated with preserved vision and reduced ocular complications, especially in the case of post‐PPV endophthalmitis caused by a highly virulent organism.

## Author Contributions

Roselind L. Ni: Writing—review and editing, writing—original draft, visualization, and investigation. Evelyn G. Miller: Writing—review and editing and visualization. Allen Chiang: Writing—review and editing, conceptualization, visualization, investigation, and supervision. Michael A. Klufas: Writing—review and editing, conceptualization, visualization, investigation, and supervision. Jonathan Perez: Writing—review and editing and visualization.

## Funding

No funding was received for this manuscript.

## Disclosure

Dr. Michael A. Klufas affirms that this manuscript is an honest, accurate, and transparent account of the study being reported; that no important aspects of the study have been omitted; and that any discrepancies from the study as planned (and, if relevant, registered) have been explained. All authors have read and approved the final version of the manuscript. Dr. Michael A. Klufas had full access to all of the data in this study and takes complete responsibility for the integrity of the data and the accuracy of the data analysis.

## Ethics Statement

Our institution does not require ethical approval for reporting individual cases. The case report complies with the Health Insurance Portability and Accountability Act and the Declaration of Helsinki.

## Consent

The patient has given verbal consent to publish this case, which contains no identifiable features.

## Conflicts of Interest

The authors declare no conflicts of interest.

## Data Availability

The data that support the findings of this study are available from the corresponding author upon reasonable request.

## References

[1] Dave V. P. , Pathengay A. , Schwartz S. G. , and Flynn H. W. , Endophthalmitis Following Pars Plana Vitrectomy: A Literature Review of incidence, Causative Organisms, and Treatment Outcomes, Clinical Ophthalmology. (2014) 8, 2183–2188, 10.2147/OPTH.S71293, 25382968. PMC4222626 25382968

[2] Clarke B. , Williamson T. H. , Gini G. , and Gupta B. , Management of Bacterial Postoperative Endophthalmitis and the Role of Vitrectomy, Survey of Ophthalmology. (2018) 63, no. 5, 677–693, 10.1016/j.survophthal.2018.02.003, 29453989. 29453989

[3] Sridhar J. , Kuriyan A. E. , Flynn H. W. , and Miller D. , Endophthalmitis Caused by *Pseudomonas aeruginosa*: Clinical Features, Antibiotic Susceptibilities, and Treatment Outcomes, Retina. (2015) 35, no. 6, 1101–1106, 10.1097/IAE.0000000000000469. 25658178

[4] Wu L. , Berrocal M. H. , Arévalo J. F. , Carpentier C. , Rodriguez F. J. , Alezzandrini A. , Farah M. E. , Roca J. A. , Maia M. , Saravia M. , Morales-Cantón V. , Graue-Wiechers F. , and Cardillo J. A. , Endophthalmitis After Pars Plana Vitrectomy, Retina. (2011) 31, no. 4, 673–678, 10.1097/IAE.0b013e318203c183. 21394065

[5] Oshima Y. , Kadonosono K. , Yamaji H. , Inoue M. , Yoshida M. , Kimura H. , Ohji M. , Shiraga F. , Hamasaki T. , and Japan Microincision Vitrectomy Surgery Study Group , Multicenter Survey With a Systematic Overview of Acute-Onset Endophthalmitis After Transconjunctival Microincision Vitrectomy Surgery, American Journal of Ophthalmology. (2010) 150, no. 5, 716–725.e1, 10.1016/j.ajo.2010.06.002, 20719299. 20719299

[6] Hu A. Y. H. , Bourges J.-L. , Shah S. P. , Gupta A. , Gonzales C. R. , Oliver S. C. N. , and Schwartz S. D. , Endophthalmitis After Pars Plana Vitrectomy, Ophthalmology. (2009) 116, no. 7, 1360–1365, 10.1016/j.ophtha.2009.01.045. 19576499

[7] McGuiness O. A. , Sivam S. , Menadue C. , Salama R. O. , Hal S. V. , Yee B. J. , and Piper A. J. , Iatrogenic Infection Associated With Positive Airway Pressure Therapy: A Review of Precedent, Epidemiology, Bioaerosols and Risk Mitigation, Sleep Medicine Reviews. (2025) 79, 102017, 10.1016/j.smrv.2024.102017, 39476478. 39476478

[8] Chen K.-J. , Sun M.-H. , Lai C.-C. , Wu W. C. , Chen T. L. , Kuo Y. H. , Chao A. N. , Hwang Y. S. , Chen Y. P. , Wang N. K. , Liu L. , and Kuo J. Z. C. , Endophthalmitis Caused by *Pseudomonas aeruginosa* in Taiwan, Retina. (2011) 31, no. 6, 1193–1198, 10.1097/IAE.0b013e3181fbce5c, 21283056. 21283056

[9] Eifrig C. W. G. , Scott I. U. , Flynn H. W. , and Miller D. , Endophthalmitis Caused by *Pseudomonas aeruginosa* , Ophthalmology. (2003) 110, no. 9, 1714–1717, 10.1016/S0161-6420(03)00572-4. 13129867

[10] Lin J. , Huang S. , Liu M. , Lin L. , Gu J. , and Duan F. , Endophthalmitis Caused by *Pseudomonas aeruginosa*: Clinical Characteristics, Outcomes, and Antibiotics Sensitivities, Journal of Ophthalmology. (2022) 2022, 1265556, 10.1155/2022/1265556, 36157680. PMC9492326 36157680

[11] Endophthalmitis Vitrectomy Study Group , Results of the Endophthalmitis Vitrectomy Study, Archives of Ophthalmology. (1995) 113, no. 12, 1479–1494, 10.1001/archopht.1995.01100120009001. 7487614

[12] Welch S. , Bhikoo R. , Wang N. , Siemerink M. J. , Shew W. , Polkinghorne P. J. , and Niederer R. L. , Better Visual Outcome Associated With Early Vitrectomy in the Management of Endophthalmitis, British Journal of Ophthalmology. (2022) 106, no. 8, 1145–1149, 10.1136/bjophthalmol-2020-316846, 33712482. 33712482

[13] Blom K. , Jørstad Ø. K. , Faber R. T. , Stene-Johansen I. , Holberg-Petersen M. , Hermansen N. O. , and Bragadóttir R. , Primary Vitrectomy or Intravitreal Antibiotics Followed by Early Vitrectomy for Acute Endophthalmitis: A Prospective Observational Study, Acta Ophthalmologica. (2023) 101, no. 1, 100–108, 10.1111/aos.15207, 35751448. PMC10084037 35751448

[14] Wang T. , Moinuddin O. , Abuzaitoun R. , Hwang M. , Besirli C. , Wubben T. J. , and Zacks D. N. , Retinal Detachment After Endophthalmitis: Risk Factors and Outcomes, Clinical Ophthalmology. (2021) 15, 1529–1537, 10.2147/OPTH.S302757, 33880012. PMC8053519 33880012

